# Phenolic Compounds from the Flowers of *Bombax malabaricum* and Their Antioxidant and Antiviral Activities

**DOI:** 10.3390/molecules201119660

**Published:** 2015-11-05

**Authors:** Yu-Bo Zhang, Peng Wu, Xiao-Li Zhang, Chao Xia, Guo-Qiang Li, Wen-Cai Ye, Guo-Cai Wang, Yao-Lan Li

**Affiliations:** 1 Institute of Traditional Chinese Medicine and Natural Products, College of Pharmacy, Jinan University, Guangzhou 510632, China; E-Mails: ybzhang99@126.com (Y.-B.Z.); yanknow@163.com (P.W.); gzleijie@gmail.com (X.-L.Z.); summerxc123456@gmail.com (C.X.); liguoqiang@jnu.edu.cn (G.-Q.L.); chyewc@gmail.com (W.-C.Y.); 2 Shenzhen Engineering Laboratory of Lingnan Herbal Resource Development and Application, Shenzhen Institute for Drug Control, Shenzhen 518057, China; 3 International Institute for Translational Chinese Medicine, Guangzhou University of Chinese Medicine, Guangzhou 510006, China

**Keywords:** *Bombax malabaricum*, phenolic compounds, structure identification, antiviral activity, antioxidant activity

## Abstract

Three new phenolic compounds **1**–**3** and twenty known ones **4**–**23** were isolated from the flowers of *Bombax malabaricum.* Their chemical structures were elucidated by spectroscopic analyses (IR, ESI-MS, HR-ESI-MS, 1D- and 2D-NMR) and chemical reactions. The antioxidant capacities of the isolated compounds were tested using FRAP and DPPH radical-scavenging assays, and compounds **4**, **6**, **8**, **12**, as well as the new compound **2**, exhibited stronger antioxidant activities than ascorbic acid. Furthermore, all of compounds were tested for their antiviral activities against RSV by the CPE reduction assay and plaque reduction assay. Compounds **4**, **10**, **12** possess *in vitro* antiviral activities, and compound **10** exhibits potent anti-RSV effects, comparable to the positive control ribavirin.

## 1. Introduction

*Bombax malabaricum* DC. (Bombacaceae) is a very common native tree in Guangdong Province of China. In the folklore of southern China its flowers are often used as a healthy food material, which is stewed in soup with meat and cooked congee with rice [1]. This flower and four other flowers from medicinal herbs are mixed as a health care tea called “five flowers tea” [2]. According to the traditional Chinese medicine theory, the flower of *B. malabaricum* is sweet and cool-natured [1], and traditionally used for the treatment of diarrhea, chronic inflammation, fever, hepatitis, and contused wounds [3]. Modern pharmacology research has shown that the *Bombax* plant possesses many biological activities, such as antioxidant [4], anti-inflammatory [5], anticancer [6], and protection of the hepatic and cardiovascular systems [7]. Phytochemical investigations have shown that flavonoids, sesquiterpenes and phenylpropanoids are the major constituents of *B. malabaricum* [8,9,10].

**Figure 1:**
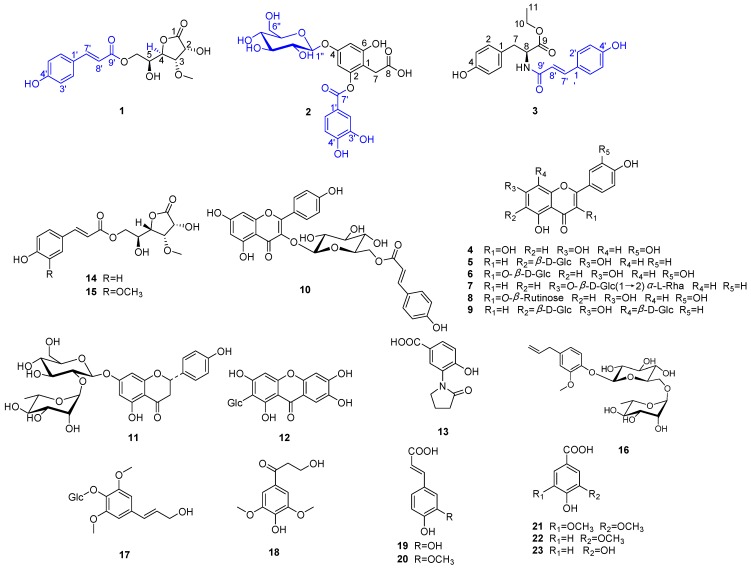
Chemical structures of **1**–**23**.

In the present work, we found that the ethanol extract of *B. malabaricum* flower possessed *in vitro* antiviral activity against respiratory syncytial virus (RSV). Furthermore, a previous study had showed that its ethanol extract displayed good antioxidant activity [4]. As a part of our continuing study on the isolation of interesting and biologically active compounds from this plant, three new phenolic compounds and 20 known ones (Figure 1) were isolated from the ethanol extract of this plant. In this paper, the isolation and structural elucidation of the new compounds are reported. In addition, the anti-RSV activities of the isolates were evaluated by cytopathic effect (CPE) and plaque reduction assays, and their antioxidant activities were tested by using ferric-reducing antioxidant power (FRAP) and 2,2-diphenyl-1-picryhydrazyl (DPPH) radical-scavenging assays.

## 2. Results and Discussion

### 2.1. Identification of Compounds **1**–**23**

**Figure 2:**
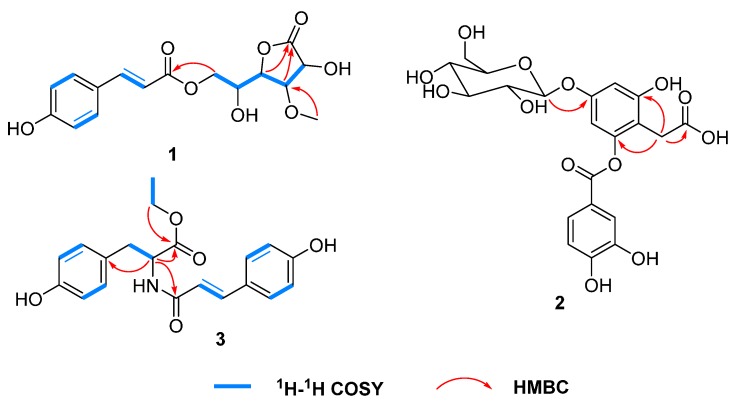
Key COSY and HMBC correlations of **1**–**3**.

Compound **1** was isolated as a colorless oil. Its molecular formula was determined as C_16_H_18_O_8_ on the basis of a HR-ESI-MS peak at *m*/*z* 361.08977 [M + Na]^+^ (calcd. for C_16_H_18_O_8_Na: 361.08939). The ^1^H-NMR spectrum of **1** showed the presence of four aromatic protons [δ_H_ 7.47 (2H, d, *J* = 8.4 Hz), 6.81 (2H, d, *J* = 8.4 Hz)], two *trans*-olefinic protons [δ_H_ 7.67 (1H, d, *J* = 15.9 Hz), 6.38 (1H, d, *J* = 15.9 Hz)] and one methoxyl group [δ_H_ 3.60 (3H, s)]. The ^13^C-NMR spectrum exhibited two carbonyls at δ_C_ 177.4 and 168.6, six aromatic carbons at δ_C_ 127.3, 131.4 × 2, 117.0 × 2, 161.5, one double bond at δ_C_ 147.2 and 115.2, and one methoxyl group at δ_C_ 61.1. Comparison of the ^13^C-NMR data of **1** with those of known compound **14** (Table S1, see Supplementary Materials) [9] showed that they were very similar, except for some differences of the chemical shifts on C-4, C-5 and C-6. These differences were about 2.0–4.7 ppm, indicating the configuration of C-4 was different from that of **14**. The planar structure of **1** was verified by ^1^H-^1^H COSY, and HMBC spectra (Figure 2). The ROESY correlations between H-2 and H-3/H-5 suggested that H-2/H-3 were on the same side and H-4 was on the other side. Thus the structure of **1** was elucidated and it was named 4-*epi*-bombalin.

Compound **2** was isolated as a colorless oil. The molecular formula of **2** was verified as C_21_H_22_O_13_ by an [M + Na]^+^ ion peak at *m*/*z* 505.09584 (calcd. for C_21_H_22_O_13_Na: 505.09526) in the HR-ESI-MS. The signals of five aromatic protons at δ_H_ 7.58 (1H, dd, *J* = 8.4, 2.2 Hz), 7.57 (1H, d, *J* = 2.2 Hz), 6.87 (1H, d, *J* = 8.4 Hz), 6.56 (1H, d, *J* = 2.2 Hz) and 6.47 (1H, d, *J* = 2.2 Hz), and one sugar at δ_H_ 4.89 (1H, d, *J* = 7.6 Hz) and 3.70–3.88 (6H, overlapped) were displayed by the ^1^H-NMR spectrum. Accordingly, the signals of two carbonyls at δ_C_ 177.7 and 166.6, one glucose moiety at δ_C_ 102.4, 78.2, 78.0, 75.0, 71.4 and 62.6, and two benzene rings at δ_C_ 103–159 were exhibited by the ^13^C-NMR spectrum. The ^1^H- and ^13^C-NMR data (Table 1) showed a number of similarities to those of 2-*O*-(3,4-dihydroxybenzoyl)-2,4,6-trihydroxyphenylacetic acid [11], except that **2** had an extra glucose and the chemical shifts of H-3 and H-5 at δ_H_ 6.27 and 6.18 shifted downfield by about 0.3 ppm to δ_H_ 6.56 and 6.47, suggesting the glucose was connected to C-4. And it was confirmed by the HMBC correlation between δ_H_ 4.89 (H-1′′) and δ_C_ 158.6 (C-4) (Figure 2). Acid hydrolysis of **2** afforded d-glucose (Figure S17, see Supplementary Materials), which was identified by HPLC analysis [12].

The coupling constant (7.6 Hz) of the anomeric proton indicated the glucose was β configuration. Compound **2** was thus identified as 2-*O*-(3,4-dihydroxybenzoyl)-2,4,6-trihydroxyphenylacetic acid 4-*O*-β-d-glucopyranoside.

Compound **3** was isolated as a colorless oil. The HR-ESI-MS of compound **3** exhibited a quasi-molecular ion peak at *m*/*z* 356.14900 [M + H]^+^ (calcd. for C_20_H_22_NO_5_: 356.14925) indicating the molecular formula was C_20_H_21_NO_5_. The IR spectrum showed the presence of NH (3357 cm^−1^) and carbonyl groups (1730, 1650 cm^−1^). The ^1^H-NMR spectrum showed two *trans*-olefinic protons at δ_H_ 7.42 (1H, d, *J* = 15.9 Hz) and 6.45 (1H, d, *J* = 15.9 Hz), two sets of aromatic protons at δ_H_ 7.38 (2H, d, *J* = 8.4 Hz), 6.78 (2H, d, *J* = 8.4 Hz) and 7.03 (2H, d, *J* = 8.4 Hz), 6.70 (2H, d, *J* = 8.4 Hz), and an ethyoxyl at δ_H_ 4.12 (2H, q, *J* = 7.1 Hz) and 1.19 (3H, t, *J* = 7.1 Hz). The ^13^C-NMR spectrum exhibited the presence of two carbonyl groups at δ_C_ 173.5 and 169.1, two benzene rings and one double bond in the region δ_C_ 116–161, and one methyl at δ_C_ 14.5. The structure of **3** was elucidated by the HMBC correlations from δ_H_ 4.12 (2H, q, *J* = 7.1 Hz, H-10) to δ_C_ 173.5 (C-9), and from δ_H_ 4.68 (1H, dd, *J* = 8.0, 6.4 Hz, H-8) to δ_C_ 38.1 (C-7), 128.9 (C-1), 169.1 (C-9′) and 173.5 (C-9) (Figure 2). The configration of natural amino acid was l, suggesting the configration of C-8 was *S*. This was confirmed by the optical rotation of **3**, which was similar to that of tyrosine (${\left[\alpha \right]}_{D}^{27}$ = −36.5) [13]. Compound **3** was previously identified as a synthetic l-tyrosine derived polymer [14] and named as *N*-[(2*E*)-3-(4-hydroxyphenyl)-1-oxo-2-propen-1-yl]-l-tyrosine ethyl ester. Here it was reported for the first time as a molecule isolated from Nature. 

**Table 1:** ^1^H- and ^13^C-NMR data of **1**–**3** (in CD_3_OD, *J* in Hz) ^a,b^.

1			2			3		
Position	δ_C_	δ_H_	Position	δ_C_	δ_H_	Position	δ_C_	δ_H_
1	177.4	-	1	111.9	-	1	128.9	-
2	73.4	4.70 d (4.5)	2	159.0	-	2	131.4	7.03 d (8.4)
3	80.2	4.16 dd (4.5, 3.6)	3	103.3	6.56 d (2.2)	3	116.4	6.70 d (8.4)
4	79.6	4.73 dd (8.1, 3.6)	4	158.6	-	4	157.6	-
5	74.3	5.35 ddd (8.1, 3.6, 3.0)	5	103.7	6.47 d (2.2)	5	116.4	6.70 d (8.4)
6	61.6	3.82 m	6	151.9	-	6	131.4	7.03 d (8.4)
1′	127.3	-	7	32.0	3.47 s	7	38.1	2.93 dd (14.0, 8.0) 3.05 dd (14.0, 6.4)
2′	131.4	7.47 d (8.4)	8	177.7	-	8	56.0	4.68 dd (8.0, 6.4)
3′	117.0	6.81 d (8.4)	1′	121.7	-	9	173.5	-
4′	161.5	-	2′	118.1	7.57 d (2.2)	10	62.4	4.12 q (7.1)
5′	117.0	6.81 d (8.4)	3′	146.5	-	11	14.5	1.19 t (7.1)
6′	131.4	7.47 d (8.4)	4′	152.6	-	1′	127.8	-
7′	147.2	7.67 d (15.9)	5′	116.1	6.87 d (8.4)	2′	130.8	7.38 d (8.4)
8′	115.2	6.38 d (15.9)	6′	124.7	7.58 dd (8.4, 2.2)	3′	116.9	6.78 d (8.4)
9′	168.6	-	7′	166.6	6.45 d (15.9)	4′	160.8	-
3-OCH_3_	61.1	3.60 s	1′′	102.4	4.89 (7.6)	5′	116.9	6.78 d (8.4)
			2′′	75.0	3.44	6′	130.8	7.38 d (8.4)
			3′′	78.0	3.43	7′	142.7	7.42 d (15.9)
			4′′	71.4	3.40	8′	117.9	6.45 d (15.9)
			5′′	78.2	3.43	9′	169.1	-
			6′′	62.6	3.88 d (12.0) 3.70 dd (12.0, 3.9)			

^a^ Signals overlapped with solvent signals; ^b^ **1**, **2** was measured at 300 MHz, **3** was measured at 400 MHz.

In addition, the other 20 known phenolic compounds (Figure 1) were identified as quercetin (**4**) [15], isovitexin (**5**) [16], isoquercitrin (**6**) [17], apigenin-7-*O*-β-neohesperidoside (**7**) [18], rutin (**8**) [19], vicenin II (**9**) [20], kaempferol-3-*O*-(6″-*O*-*E*-*p*-coumaroyl)-β-d-glucopyranoside (**10**) [21], naringenin (**11**) [22], mangiferin (**12**) [23], 4-hydroxy-5-(2-oxo-1-pyrrolidinyl)-benzoic acid (**13**) [24], bombalin (**14**) [9], amurenlactone A (**15**) [25], eugenyl β-rutinoside (**16**) [26], syringin (**17**) [27], 3-hydroxy-1-(4-hydroxy-3,5-dimethoxyphenyl)-1-propanone (**18**) [28], caffeic acid (**19**) [29], ferulic acid (**20**) [30], syringic acid (**21**) [31], vanillic acid (**22**) [32] and protocatechuic acid (**23**) [33], respectively, by comparison of their spectroscopic data with those of previous literatures.

### 2.2. Antioxidant Activities

The ethanol extract of *B. malabaricum* flower was reported to have good antioxidant activity [4]. Among the compounds isolated from the ethanol extract of *B. malabaricum* flower, compounds **2**, **4**, **6**, **8** and **12** showed potent antioxidant activities under both DPPH and FRAP assays (Table 2) [34,35]. Compound **2**, a new compound from the flower of *B. malabaricum*, showed a strong radical scavenging activity with SC_50_ value of 11.3 ± 1.6 μM, while the SC_50_ value of ascorbic acid of 16.3 ± 0.7 μM. In addition, FRAP assay also gave the same result that this new compound possessed potent antioxidant activity. Regarding the structure of compound **2**, the 3,4-dihydroxyphenyl moiety and phenolic hydroxyls were considered as the antioxidant functional groups [36]. Compounds **4**, **6**, **8** and **12** also exhibited potent DPPH radical-scavenging activities with SC_50_ values of 6.0 ± 0.3, 10.8 ± 1.2, 9.6 ± 0.7 and 14.5 ± 2.3 μM, respectively. According to the structures of these four compounds, the ring B of catechol groups may play an important role in their antioxidant activities [7], while the number of the free hydroxyl also has a positive effect on the antioxidant activities [7].

**Table 2:** Antioxidant activity of compounds **1**–**23**.

Compounds	DPPH SC_50_ (μM) ^a^	FRAP Value (μM) ^b^
**1**	400.8 ± 25.9	28.8 ± 0.9
**2**	11.3 ± 1.6	336.9 ± 17.0
**3**	>500 ^c^	n.d. ^d^
**4**	6.0 ± 0.3	139.5 ± 5.0
**5**	487.1 ± 25.6	18.2 ± 0.5
**6**	10.8 ± 1.2	367.1 ± 23.7
**7**	>500	n.d.
**8**	9.6 ± 0.7	379.6 ± 5.2
**9**	>500	n.d.
**10**	265.4 ± 12.7	19.6 ± 0.6
**11**	>500	n.d.
**12**	14.5 ± 2.3	371.3 ± 14.8
**13**	>500	n.d.
**14**	380.4 ± 22.7	35.8 ± 1.0
**15**	450.8 ± 16.9	47.3± 1.2
**16**	>500	n.d.
**17**	>500	n.d.
**18**	>500	n.d.
**19**	21.3 ± 0.4	111.3 ± 1.5
**20**	27.5 ± 1.3	102.5 ± 3.9
**21**	393.9 ± 11.8	n.d.
**22**	147.0 ± 8.1	12.3 ± 0.6
**23**	86.1 ± 5.8	52.3 ± 1.2
Ascorbic acid	16.3 ± 0.7	417.4 ± 9.8

^a^ SC_50_ is expressed as the concentration of sample needed to scavenge 50% of DPPH radical; data are represented as mean ± SD; ^b^ The FRAP value is the concentration of sample (μM) giving an absorbance increase equivalent to 1 mM Fe^2+^ solution; data are represented as mean ± SD; ^c^ The SC_50_ value of sample is higher than 500 μM; ^d^ n.d. Not detectable.

### 2.3. Anti-RSV Activities

In this work, we found that the ethanol extract of *B. malabaricum* flower possessed *in vitro* anti-RSV activity with an IC_50_ value of 50.0 μg/mL. Therefore, we investigated the *in vitro* anti-RSV activities of the compounds isolated from this extract. First of all, we evaluated the anti-RSV effects of the compounds with the CPE reduction assay, and found that compounds **4** [37], **10**, **12** possessed this effect to a different extent. The anti-RSV activities of these three compounds were further confirmed by the plaque reduction assay which is a quantitative method. As shown in Table 3, compounds **4**, **10**, **12** possessed *in vitro* antiviral activities against RSV with IC_50_ values of 20.0 ± 0.6, 6.3 ± 0.2 and 40.0 ± 0.7 μM, and SI values of 12.9, >79.3, >12.5, respectively. Among the active compounds, kaempferol-3-*O*-(6″-*O*-*E*-*p*-coumaroyl)-β-d-glucopyranoside (**10**) showed potent anti-RSV activity comparable to the positive drug ribavirin. In our previous studies, we have found that caffeoyl acid derivatives from natural medicines had potent anti-RSV activities [36,38,39,40]. Compound **10** is a flavonoid glycoside with a *cis*-coumaroyl connection. These results suggest (di)hydrocinnamoyl might be the active functional groups providing potent antiviral activity against RSV. Besides, mangiferin (**12**), a main constituent of the flower of *B. malabaricum*, also demonstrated anti-RSV activity. The anti-RSV activities of compounds **10** and **12** are reported for the first time.

**Table 3:** Anti-RSV activity of the active compounds (*n* = 3).

Compounds	IC_50_/μM ^a^	CC_50_/μM ^b^	SI ^c^
**4**	20.0 ± 0.6	258.6 ± 7.9	12.9
**10**	6.3 ± 0.2	>500.0	>79.3
**12**	40.0 ± 0.7	>500.0	>12.5
Ribavirin	10.0 ± 1.3	255.9 ± 8.2	25.6

^a^ IC_50_ was detected by plaque reduction assay after the screening with CPE reduction assay; data are expressed as mean ± SD; ^b^ CC_50_ was tested by MTT assay; data were expressed as mean ± SD; ^c^ SI value equals to CC_50_/IC_50_.

## 3. Experimental Section 

### 3.1. General Procedures

Melting points were determined on an X-5 micro-melting point detector (Tech, Beijing, China). Optical rotations were measured using a JASCO P-1020 polarimeter (JASCO, Hachioji-shi, Tokyo, Japan). UV spectra were recorded on a JASCO V-550 UV/VIS spectrophotometer (JASCO). IR spectra were determined using a JASCO FT/IR-480 plus spectrophotometer with KBr pellets (JASCO). NMR spectra were recorded on a Bruker AV 300 or 400 MHz spectrometer (Bruker, Faellanden, Switzerland) with TMS as internal standard. ESI-MS data were determined by a Finnigan LCQ Advantage Max mass spectrometer (Thermo Electron, Billerica, MA, USA). HR-ESI-MS data were obtained by an Agilent 6210 LC/MSD TOF mass spectrometer (Agilent, Santa Clara, CA, USA). A Dionex chromatograph was used for analytical HPLC with a P680 pump, a PDA-100 photodiode array detector, and a 5C_18_-MS-II column (Cosmosil, 4.6 × 250 mm, 5 μm, Nacalai Tesque, Kyoto, Japan). A Varian ProStar 210 chromatograph equipped with a Varian-306 pump, a Varian UV/VIS-152 detector, and a 5C_18_-MS-II column (Cosmosil, 10 × 250 mm, 5 μm, Nacalai Tesque, Kyoto, Japan) was used for preparative HPLC. D101 macroporous resin (Mitsubishi Chemical Corporation, Tokyo, Japan), Silica gel (200−300 mesh, Qingdao Marine Chemical Inc., China), Sephadex LH-20 (Pharmacia, Sweden) and ODS (Merck, Darmstadt, Germany) were used for column chromatography (CC). The precoated silica gel plates (GF_254_, Yantai, China) were used for Thin-layer chromatography (TLC). All reagents were purchased from Tianjin Damao Chemical Company (Tianjin, China). 3-(4,5-dimethylthiazol-2-yl)-2,5-diphenyltetrazolium bromide (MTT), l-ascorbic acid, 2,2-diphenyl-1-picrylhydrazyl (DPPH), and 2,4,6-tris(2-pyridyl)-s-triazine (TPTZ) were purchased from Sigma (St. Louis, MO, USA).

### 3.2. Plant Materials

The flower of *Bombax malabaricum* was collected in Guangzhou City, Guangdong Province of China, in May of 2010. The plant was authenticated by Prof. Guang-Xiong Zhou, College of Pharmacy, Jinan University. A voucher specimen (No. 2010051520) was deposited in the Institute of Traditional Chinese Medicine & Natural Products, Jinan University, Guangzhou, China.

### 3.3. Extraction and Isolation

The powdered flower of *Bombax malabaricum* (6.2 kg) was extracted by reflux for 3 times with 95% ethanol (20 L) The solution was evaporated *in vacuo* to give a residue (956.0 g) which was suspended in water and partitioned with petroleum ether and ethyl acetate, respectively. The ethyl acetate-soluble part (72.0 g) was subjected to silica gel column chromatography (10 cm × 40 cm, 200–300 mesh, 1.0 kg) eluting with chloroform/methanol (CHCl_3_/CH_3_OH, 100:0→0:100, *v*/*v*) while monitoring by TLC (CHCl_3_/CH_3_OH, 80:20, *v*/*v*) to afford seven fractions A–G. Fraction B (5.3 g) was further separated by silica gel column chromatography (2.5 × 80 cm, 200.0 g) with CHCl_3_/CH_3_OH (98:2→70:30, *v*/*v*) and Sephadex LH-20 with CHCl_3_/CH_3_OH (50/50, *v*/*v*) as eluents to yield compounds **19** (9.0 mg), **20** (10.5 mg), **21** (5.8 mg), **22** (11.2 mg) and **23** (40.5 mg). Fraction D (3.2 g) was further separated by silica gel column chromatography (2.5 × 80 cm, 180.0 g) with CHCl_3_/CH_3_OH (98:2→80:20, *v*/*v*) to yield compounds **10** (8.0 mg), **17** (8.2 mg) and **18** (25.9 mg). Fraction H (2.9 g) was purified by Sephadex LH-20 column with CHCl_3_/CH_3_OH (50:50, *v*/*v*) and preparative HPLC with CH_3_OH/H_2_O (60:40, *v*/*v*) to yield compounds **3** (10.5 mg), **4** (81.0 mg), and **13** (7.0 mg). The water-soluble part (790.0 g) was applied to a D101 macroporous resin column (20 cm × 120 cm, 10 kg) eluted with water, 10%, 30%, 60% and 95% ethanol, respectively. The 30% ethanol fraction (47.4 g) was subjected to silica gel column chromatography eluting with CHCl_3_/CH_3_OH (95:5→70:30, *v*/*v*) to afford five fractions (1–5). Fraction 2 (3.1 g) was further separated by an ODS column and a preparative HPLC to yield compounds **8** (40.1 mg), **9** (46.0 mg), **11** (450.0 mg), **12** (560.0 mg), and **16** (21.8 mg). Fraction 4 (109.6 mg) was further purified on a Sephadex LH-20 column with CH_3_OH and preparative HPLC with CH_3_OH/H_2_O (45:55, *v*/*v*) to yield compounds **1** (6.8 mg), **2** (7.8 mg), **14** (7.9 mg) and **15** (9.3 mg). The 60% ethanol fraction (14.2 g) was purified by ODS column chromatography and preparative HPLC (CH_3_OH/H_2_O, 50:50, *v*/*v*) to yield compounds **5** (45.0 mg), **6** (50.1 mg) and **7** (4.5 mg).

### 3.4. Compound Characterization

*4-epi-Bombalin* (**1**): colorless oil; ${\left[\alpha \right]}_{D}^{27}$ −17.9 (*c* = 0.26, CH_3_OH); UV (CH_3_OH) λ_max_ (log *ε*): 202 (3.46), 314 (3.58) nm; IR (KBr) υ_max_: 3425, 1774, 1707, 1604, 1508, 1456, 1262 cm^−1^; ^1^H-NMR (300 MHz, CD_3_OD) and ^13^C-NMR (75 MHz, CD_3_OD) see Table 1; HR-ESI-MS (positive ion mode) *m*/*z* 361.08977 [M + Na]^+^ (calcd. for C_16_H_18_O_8_Na: 361.08939).

*2-O-(3,4-Dihydroxybenzoyl)-2,4,6-trihydroxyphenylacetic acid* *4-O-β-*d*-glucopyranoside* (**2**): colorless oil; UV (CH_3_OH) λ_max_ (log ε): 265 (3.31), 301 (3.09) nm; IR (KBr) υ_max_: 3446, 1541, 1456, 1077 cm^−1^; ^1^H-NMR (300 MHz, CD_3_OD) and ^13^C-NMR (75 MHz, CD_3_OD) see Table 1; HR-ESI-MS (positive ion mode) *m*/*z* 505.09584 [M + Na]^+^ (calcd. for C_21_H_22_O_13_Na: 505.09526).

*N-[(2E)-3-(4-Hydroxyphenyl)-1-oxo-2-propen-1-yl]-*l*-tyrosine ethyl ester* (**3**): colorless oil; ${\left[\text{α}\right]}_{\text{D}}^{27}$ −31.2 (*c* = 0.67, CH_3_OH); UV (CH_3_OH) λ_max_ (log *ε*): 228 (4.06), 310 (4.22) nm; IR (KBr) υ_max_: 3357, 2925, 1651, 1448, 1109, 828 cm^−1^; ^1^H-NMR (400 MHz, CD_3_OD) and ^13^C-NMR (100 MHz, CD_3_OD) see Table 1; HR-ESI-MS (positive ion mode) *m*/*z* 356.14900 [M + H]^+^ (calcd. for C_20_H_22_NO_5_: 356.14925).

### 3.5. Acid Hydrolysis and Sugar Analysis of **2**

Compound **2** (3.0 mg) was dissolved in 10 mL 2N HCl and heated at 80 °C for 2 h. The mixture was evaporated to dryness, and the residue was suspended in water and partitioned with dichloromethane. The aqueous phase was concentrated *in vacuum*, anhydrous pyridine (1.0 mL) and l-cysteine methyl ester hydrochloride (4.0 mg) were added, and the mixture was heated at 60 °C for 1 h. After the reaction mixture was evaporated to dryness, *o*-tolyl isothiocyanate (10 µL) was then added, and the mixture was heated at 60 °C for 1 h. The reaction mixture was directly analyzed by an Agilent 1260 HPLC system (Agilent Technologies Inc., Santa Clara, CA, USA) equipped with a photodiode array detector and a Capcell pak C_18_ column (4.6 × 250 mm, 5 μm, Cosmosil, Nacalai Tesque, Kyoto, Japan) at 25 °C with isocratic elution of 25% CH_3_CN in 0.1% formic acid solution for 40 min at a flow rate of 0.8 mL/min. The injection volume was 10 µL and peaks were detected at 250 nm. The standards d-glucose and l-glucose were treated by the same reaction and chromatographic conditions. As a result, d-glucose from the hydrolyzate of **2** was detected by the same retention time of standard sugar derivatives.

### 3.6. Antioxidant Assay

FRAP assays of compounds were estimated in triplicate according to our previous report [36]. TPTZ (10 mM) was dissolved into 40 mM HCl. FRAP reagent was prepared as required by mixing 25 mL of 0.3 M acetate buffer, 2.5 mL of 10 mM TPTZ solution and 2.5 mL of 20 mM FeCl_3_ solution. 20 μL of sample (100 μM) and 180 μL of FRAP reagent were added to a 96-well microplate. The mixtures were vortexed for 1 min and incubated for 5 min in the dark at room temperature. And then, the absorbance was detected at 593 nm with a multi-mode detection microplate reader. FeSO_4_·7H_2_O solution at different concentrations (0.15–1.5 mM) were used to establish a calibration curve. Ascorbic acid was used as the positive group. The FRAP assay results were expressed as the concentration of sample (μM) giving an absorbance increase equivalent to 1 mM Fe^2+^ solution.

The antioxidant activities were also determined by the scavenging activity of stable DPPH free radicals [36]. In a 96-well microplate, 100 μL of DPPH solution (200 μM in ethanol) was added to 100 μL of the tested compound at final concentrations (0–500 μM) in ethanol. The mixtures were shaken adequately and considered to stand for 30 min in the dark. The absorbance of the mixture was detected at 517 nm with a multi-mode detection microplate reader, and ascorbic acid was used as the positive control. The scavenging capacity of DPPH was calculated in the following way: scavenging activity (%) = 100 × (A_control_ – A_sample_)/A_control_, A_control_ is absorbance of control, A_sample_ is absorbance of sample. The concentration of sample scavenged 50% of DPPH radical was defined as the SC_50_ value.

### 3.7. Cell and Virus

Human larynx epidermoid carcinoma (HEp-2, ATCC No.: CCL-23) cell and human respiratory syncytial virus Long (ATCC No.: VR-26) strain, which were purchased from Medicinal Virology Institute, Wuhan University, China. The RSV Long strain was grown and titered in HEp-2 cells. The HEp-2 cells were cultured in Dulbecco’s modified Eagle’s medium (DMEM, Gibco, Gaithersburg, MD, USA) supplemented with 10% fetal bovine serum (FBS), 2 mM l-glutamine and 100 U/mL penicillin & streptomycin solution (Sigma, St. Louis, MO, USA). The antiviral and cytotoxic assays were tested in the medium only contained 2% FBS. Ribavirin (Sigma) was used as the positive control in the anti-RSV tests. All the cells were cultured at 37 °C in a humidified atmosphere of 5% CO_2_ (*v*/*v*).

### 3.8. Anti-RSV Activities

CPE reduction assay was adopted to screen the anti-RSV activities of the isolated compounds as described in the previous reports [36,38]. First of all, the cytotoxic activities of the ethanol extract and isolates on host cells were observed under a light microscope (DP70 Olympus, Melville, NY, USA). The maximal non-cytotoxic concentration (MNCC) of the sample was defined as the maximal concentration of the sample that did not exert toxic effect (0% CPE) under microscopic monitoring. Then, the antiviral activities of the samples were tested in the beginning concentrations of their MNCCs. Briefly, 100 μL of 100 TCID_50_ virus suspension and a serial two-fold diluted samples were added into a 96-well microplate containing confluent cell monolayer. The medium and virus suspension without sample were added as cell and virus controls, respectively. The 96-well microplate was incubated for 3–4 days. The virus-induced CPE were observed under light microscopy in comparison with the virus control and cell control.

The samples showing anti-RSV activities in CPE assay were further determined by plaque reduction assay [38]. HEp-2 cells were inoculated in 24-well plate for 24 h. The virus suspension with 60–80 plaque forming unit (PFU) and two-fold diluted samples were added a 24-well microplate containing confluent cell monolayer. The medium and virus suspension without sample were added as the cell and virus controls, respectively. The medium was inspirited by intermittent shaking at 15 min intervals for 2 h. The cell monolayers were washed twice with PBS, and then covered with agarose overlay medium. After the agarose solidified, a serial two-fold diluted samples and controls were added to the corresponding wells, respectively. And then, the plates were incubated for 4–5 days to form RSV plaques. The cells were fixed with 10% formalin and stained by 1% crystal violet. The number of plaques was counted. In the assay, half of the maximal concentration inhibiting the RSV-induced plaque formation by the sample was defined as the IC_50_ value.

### 3.9. 3-(4,5-Dimethylthiazol-2-yl)-2,5-diphenyl tetrazolium Bromide (MTT) Assay

The cytotoxicity of the anti-RSV active compounds on HEp-2 cells was detected by MTT assay in 96-well plate (Corning, Corning, NY, USA) [38]. In brief, serial two-fold dilutions of samples were added to confluent HEp-2 cell monolayers, and the medium without the sample was used as cell control. After incubation for 72 h, medium was replaced by 30 μL of the MTT solution (Sigma), and the cells were further incubated for another 4 h to allow MTT formazan formation. Then the medium was replaced by DMSO (200 μL) in each well to dissolve the formazan crystals. The optical densities (OD) values were detected by a microplate reader (Thermo Scientific, Waltham, MA, USA) at 570 nm. Each assay was performed three times, and calculated the concentration giving 50% cytotoxic concentration (CC_50_). The 50% of the sample was calculated by regression analysis of the dose-response curve generated from the OD values.

## 4. Conclusions 

In summary, three new and twenty known phenolic compounds were isolated and identified from the flower of *B. malabaricum*. The chemical structures of three new compounds **1**–**3** were identified by extensive spectroscopic methods and chemical reactions. All the compounds were tested for their antioxidant and antiviral activities *in vitro*. The results revealed that compounds **2**, **4**, **6**, **8** and **12** had potent antioxidant activities, and compounds **4**, **10**, **12** possessed moderated to strong anti-RSV effects. Compound **10**, a flavonoid glycoside with hydrocinnamoyl substitution, showed potent anti-RSV activity comparable to the positive control ribavirin. Compound **2**, a new compound, exhibited more potent antioxidant activity than the positive control ascorbic acid. Compound **12**, a main constituent in the flower of *B. malabaricum*, demonstrated both antiviral and antioxidant activities. Our study provides partial scientific support for the folk uses of *B. malabaricum* flowers.

## Supplementary Materials

The ^1^H- and ^13^C-NMR data of **14**, HR-ESI-MS, 1D-NMR, 2D-NMR spectra of compounds **1**–**3**, as well as HPLC analysis spectrum of sugar derivatives of **2** can be accessed at: http://www.mdpi.com/1420-3049/20/11/19660/s1.
