# Supplementary material for: Phenolic Compounds from the Flowers of Bombax malabaricum and Their Antioxidant and Antiviral Activities
Source: Molecules. 2015 Nov 5;20(11):19947–57. doi: 10.3390/molecules201119660 (PMC6331941; doi:10.3390/molecules201119660)
Supplement: Supplementary file 1 [file molecules-20-19660-s001.pdf]

molecules

Supplementary Informations

Table S1. NMR data of **14** (bombalin) (in CD_3OD, *J* in Hz).

Position	14	
	δ_H	δ_C
1	-	177.5
2	4.65 (1H, d, 4.5)	73.0
3	4.11 (1H, dd, 4.5, 3.6,)	80.2
4	4.47 (1H, dd, 8.0, 3.6,)	81.6
5	4.19 (1H, dd, 8.0, 4.4,)	69.6
6	4.31 (2H, m)	65.8
1′	-	127.3
2′	7.47 (1H, d, 8.5)	131.4
3′	6.81 (1H, d, 8.5)	117.0
4′	-	161.5
5′	6.81 (1H, d, 8.5)	117.0
6′	7.47 (1H, d, 8.5)	131.4
7′	7.68 (1H, d, 15.9)	147.2
8′	6.38 (1H, d, 15.9)	114.9
9′	-	169.1
3-OCH_3	3.58 (3H, s)	61.0

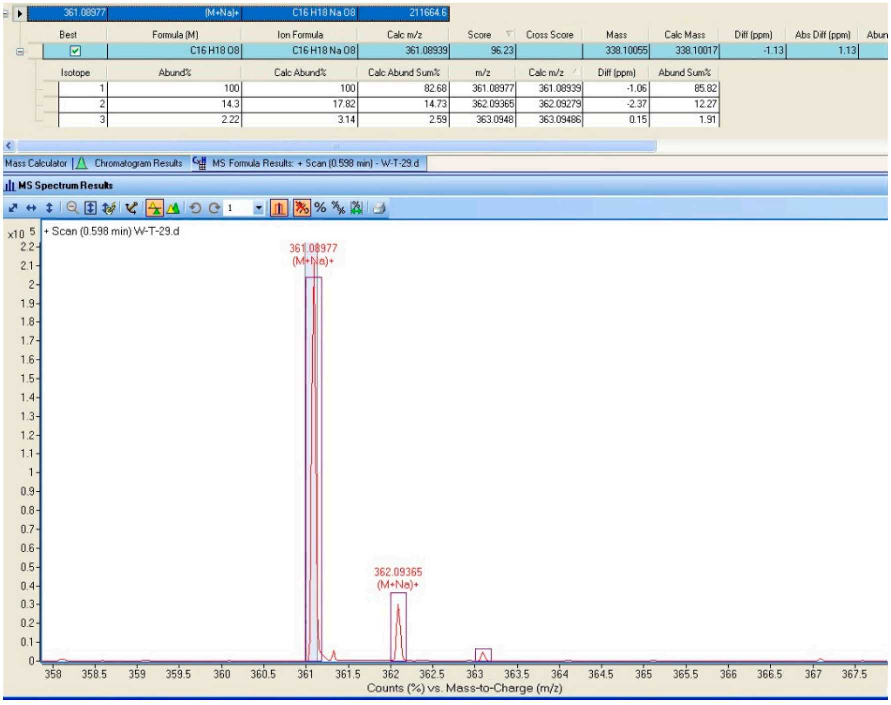

Figure S1. HR-ESI-MS spectrum of **1** (4-*epi*-bombalin).

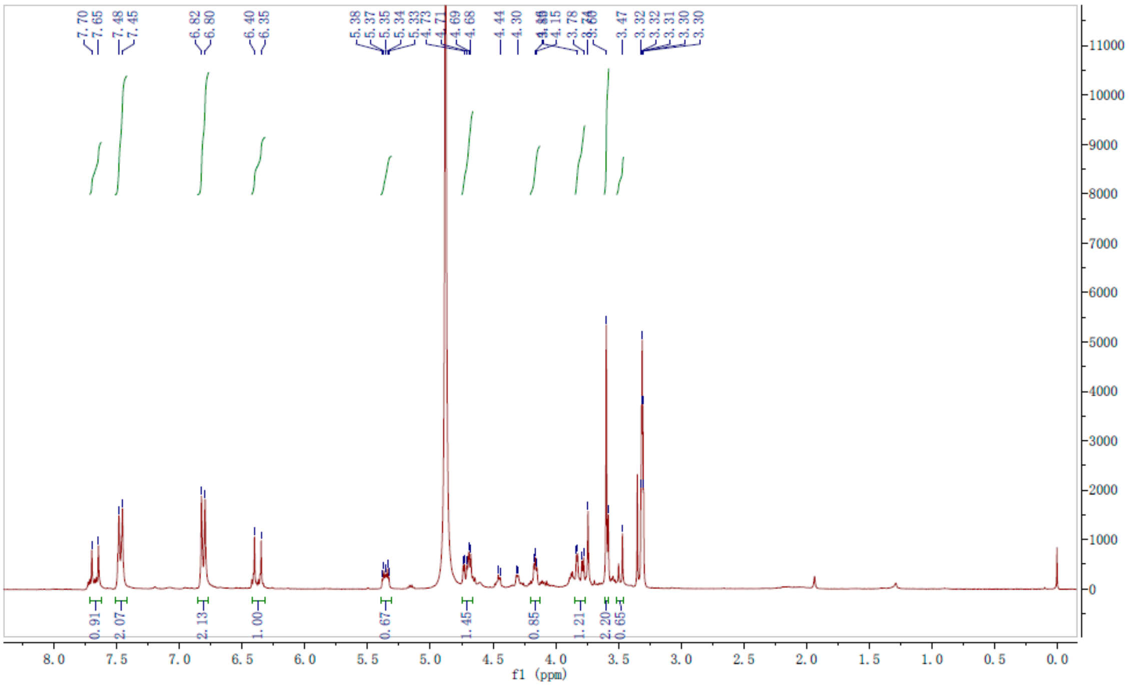

Figure S2. ^{1}H-NMR spectrum of **1** in CD_3OD.

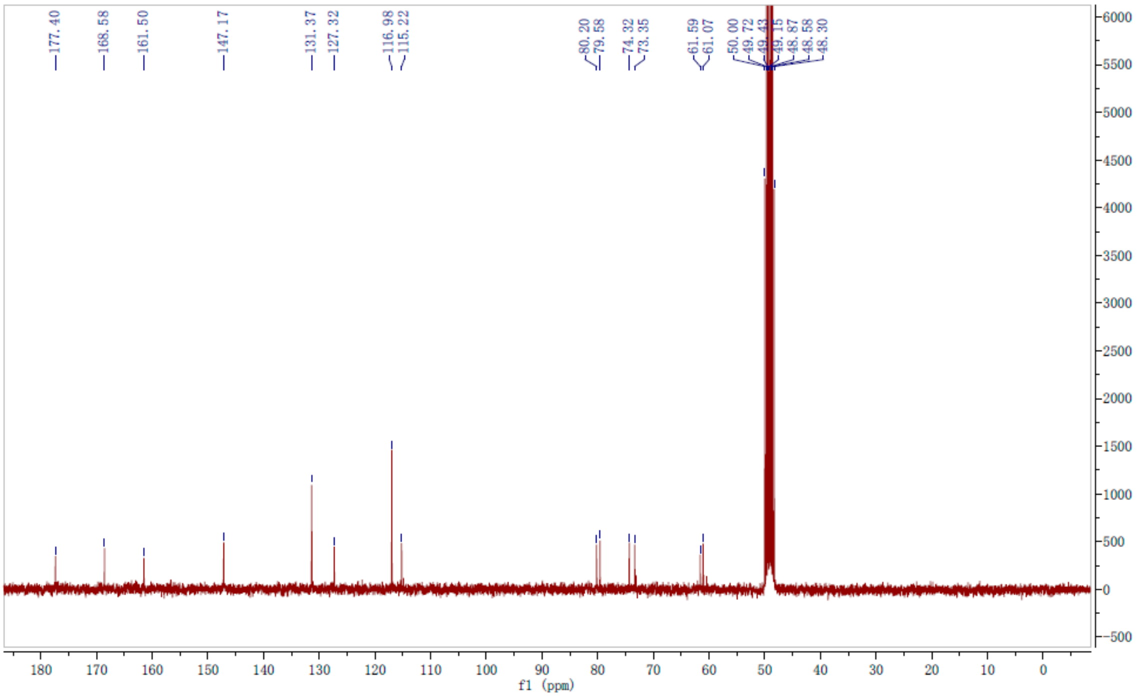

Figure S3. ^{13}C-NMR spectrum of **1** in CD_3OD.

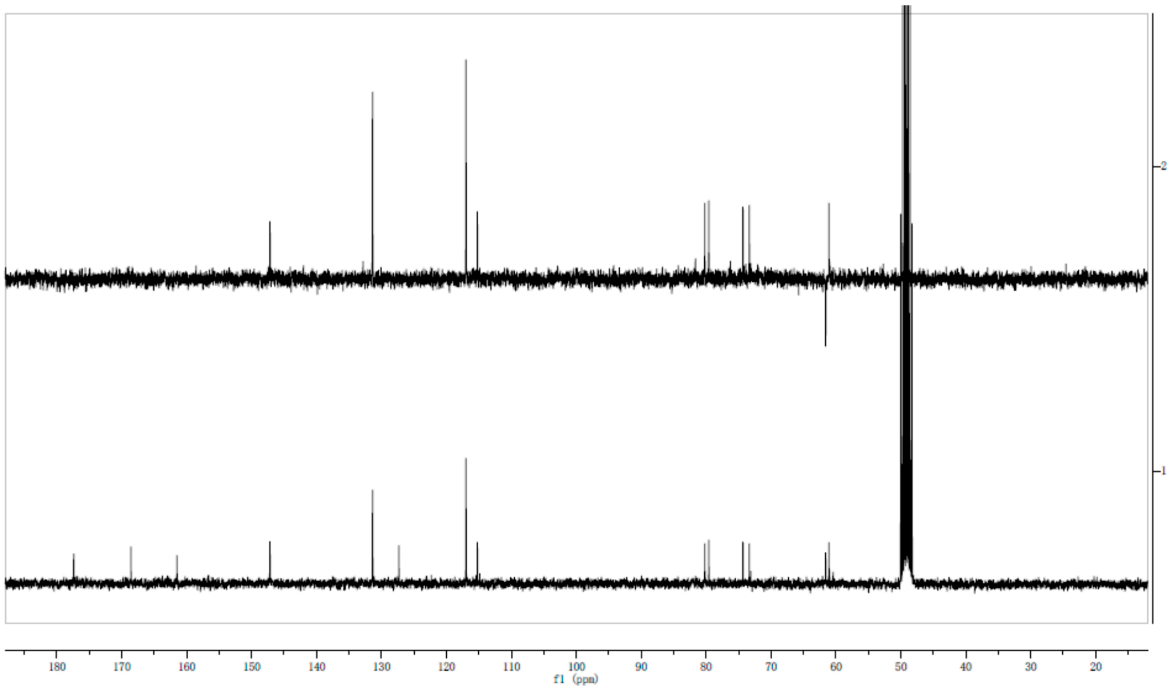

Figure S4. DEPT spectrum of **1** in CD_3OD.

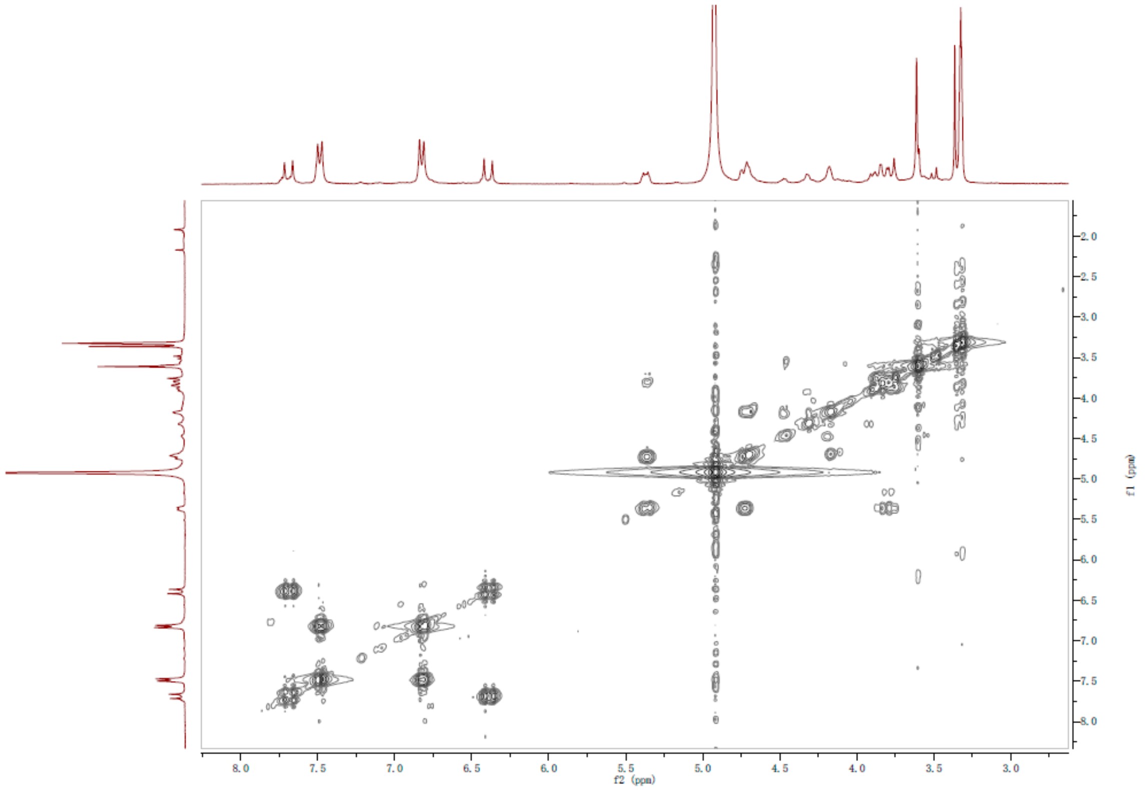

Figure S5. ^1H-^1H COSY spectrum of **1** in CD_3OD.

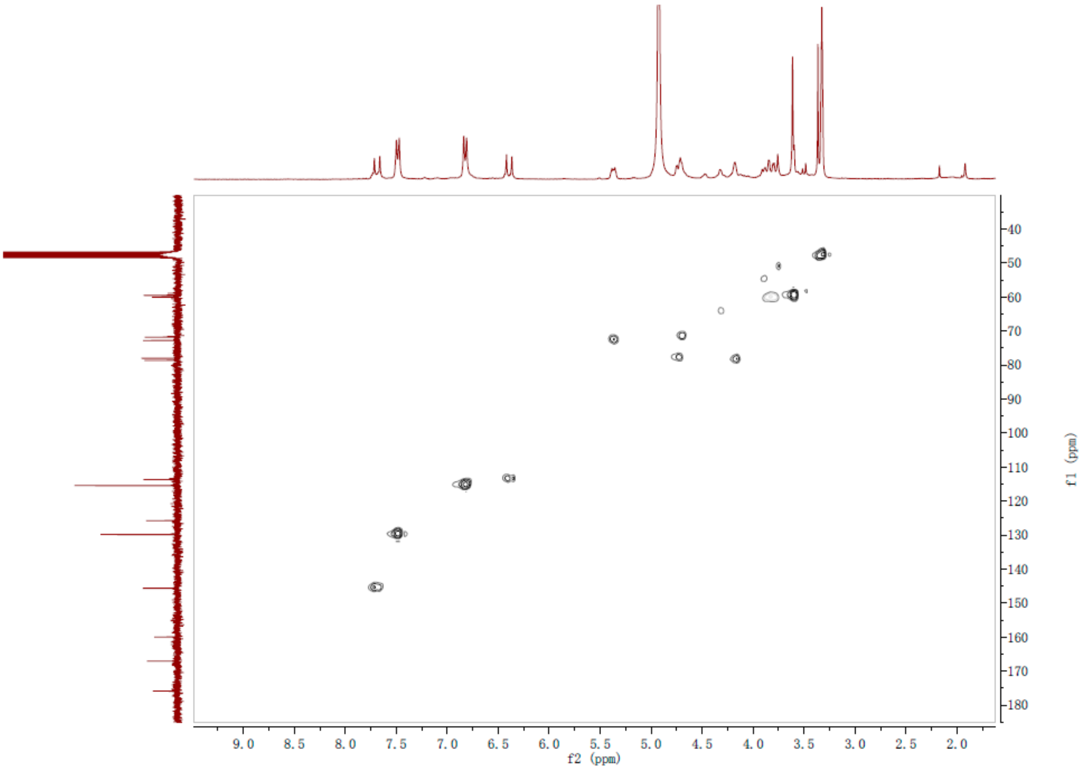

Figure S6. HSQC spectrum of **1** in CD3OD.

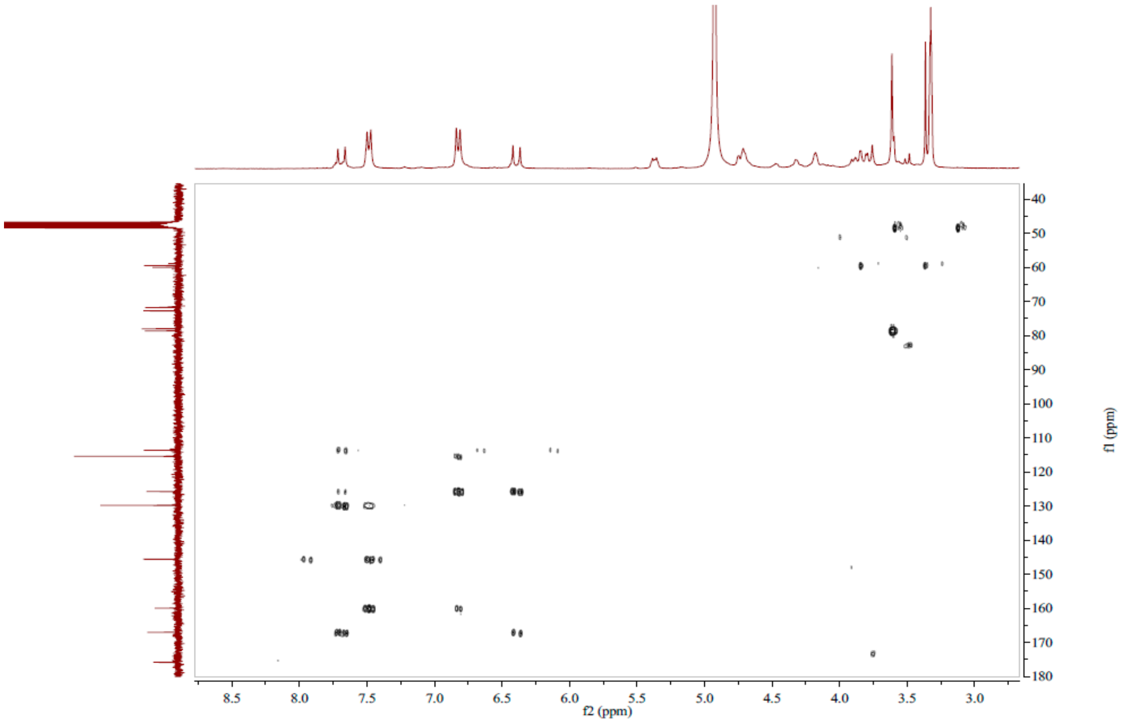

Figure S7. HMBC spectrum of **1** in CD3OD.

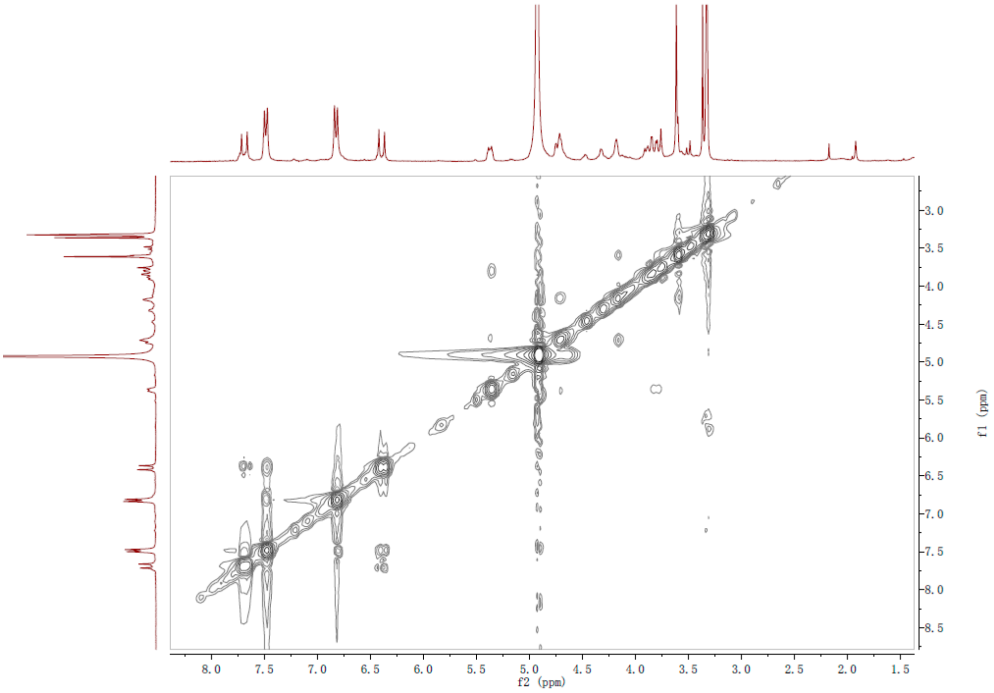

Figure S8. ROESY spectrum of **1** in CD_3OD.

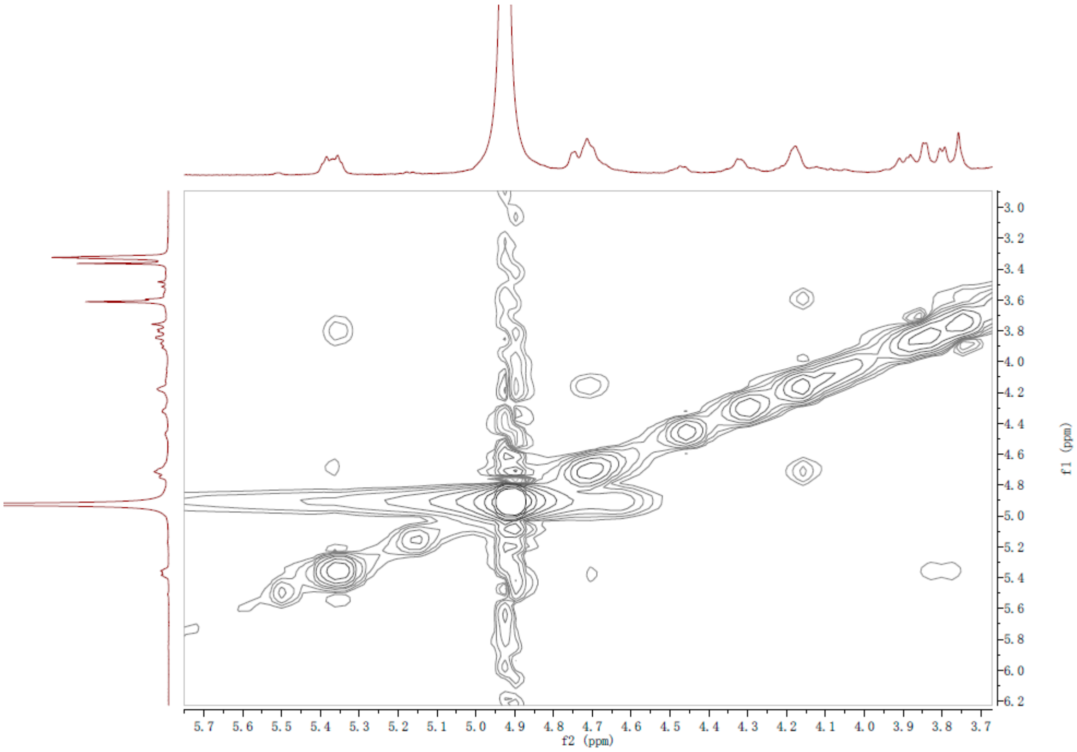

Figure S9. Enlarged ROESY spectrum of **1**.

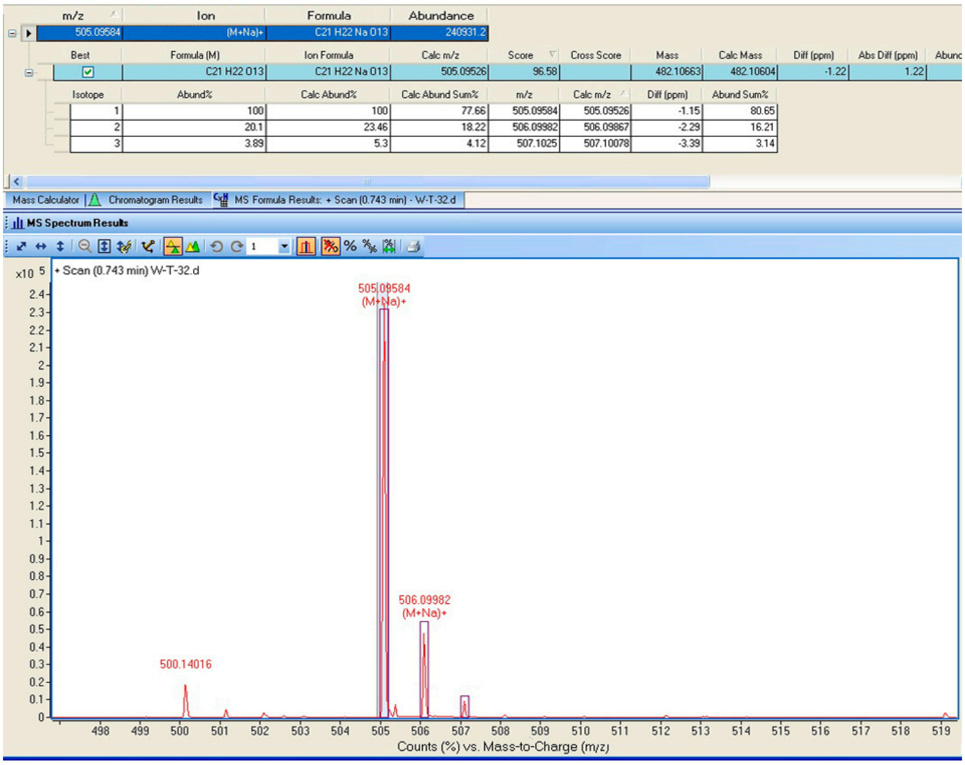

Figure S10. HR-ESI-MS spectrum of **2** (2-*O*-(3,4-dihydroxybenzoyl)-2,4,6-trihydroxyphenylacetic acid 4-*O*-β-D-glucopyranoside).

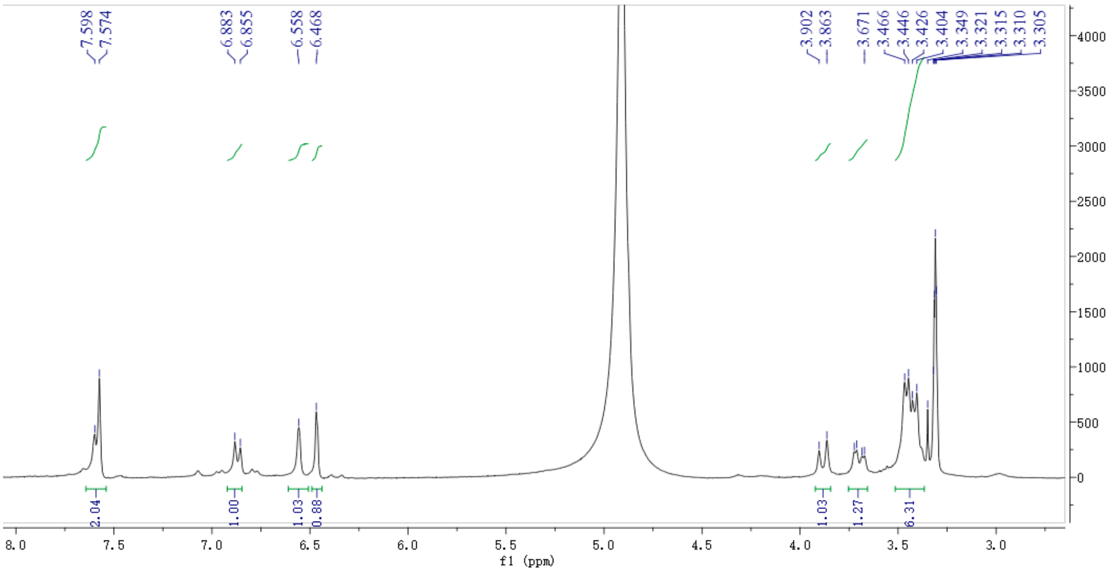

Figure S11. ^{1}H-NMR spectrum of **2** in CD_3OD.

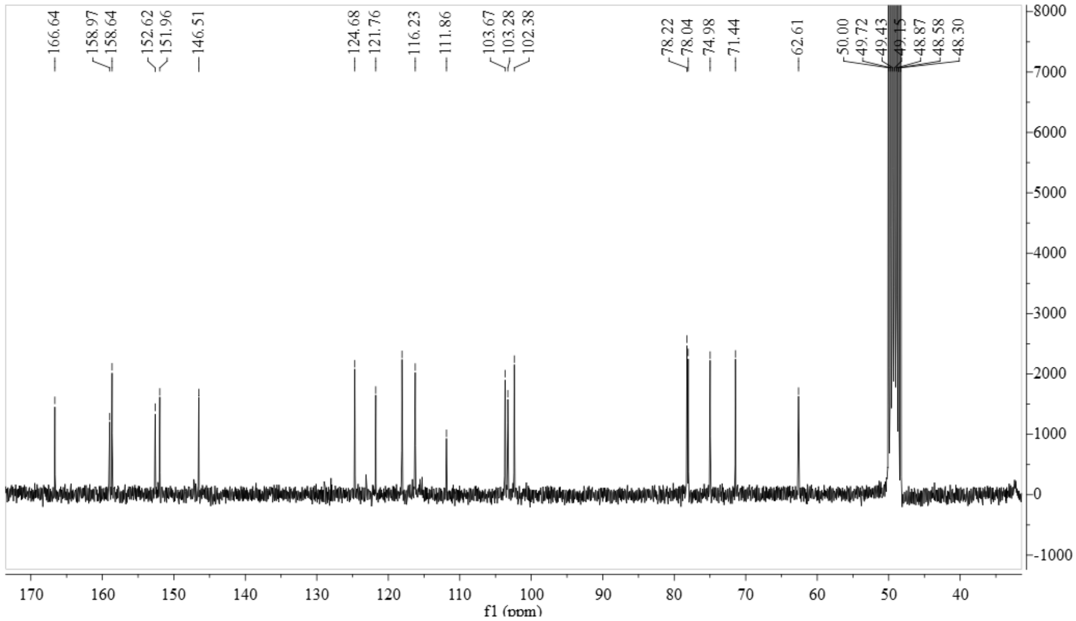

Figure S12. ^{13}C-NMR spectrum of **2** in CD_3OD.

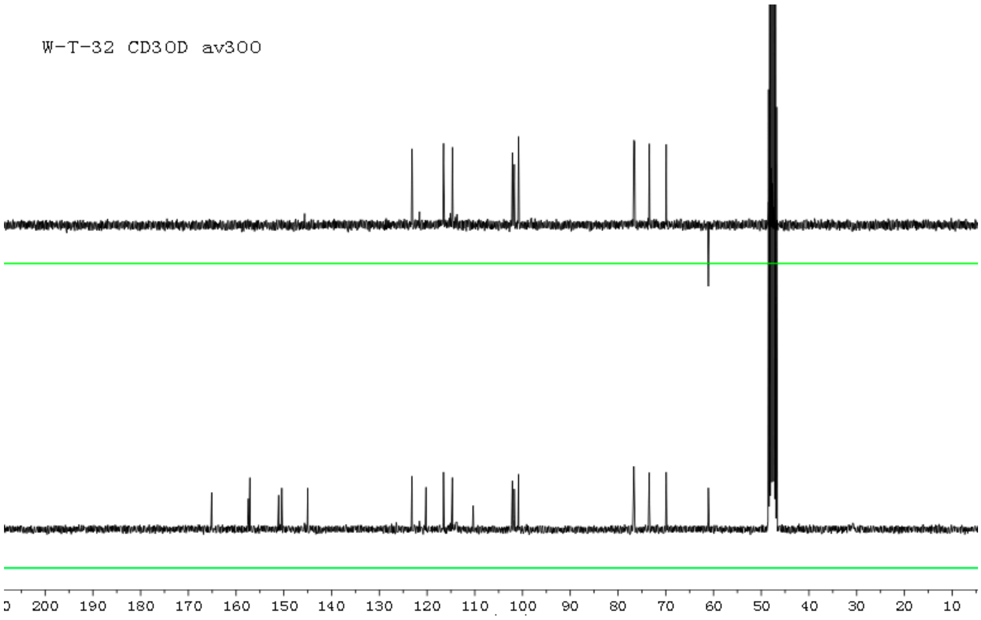

Figure S13. DEPT spectrum of **2** in CD_3OD.

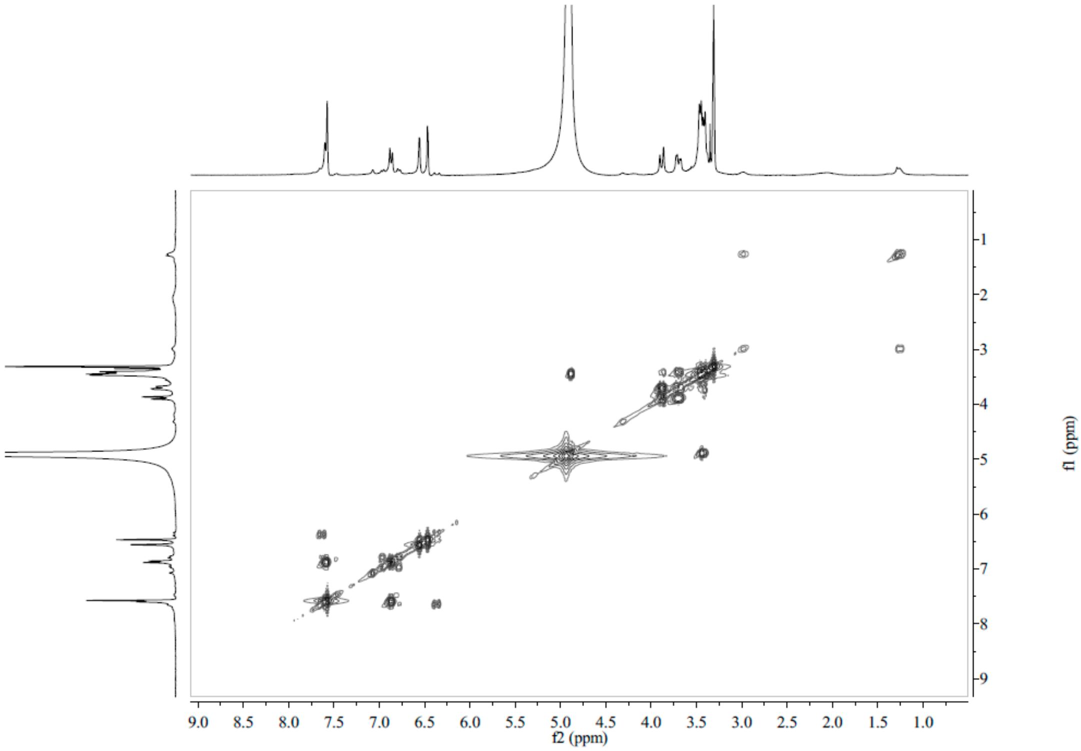

Figure S14. ^{1}H-^{1}H COSY spectrum of **2** in CD_3OD.

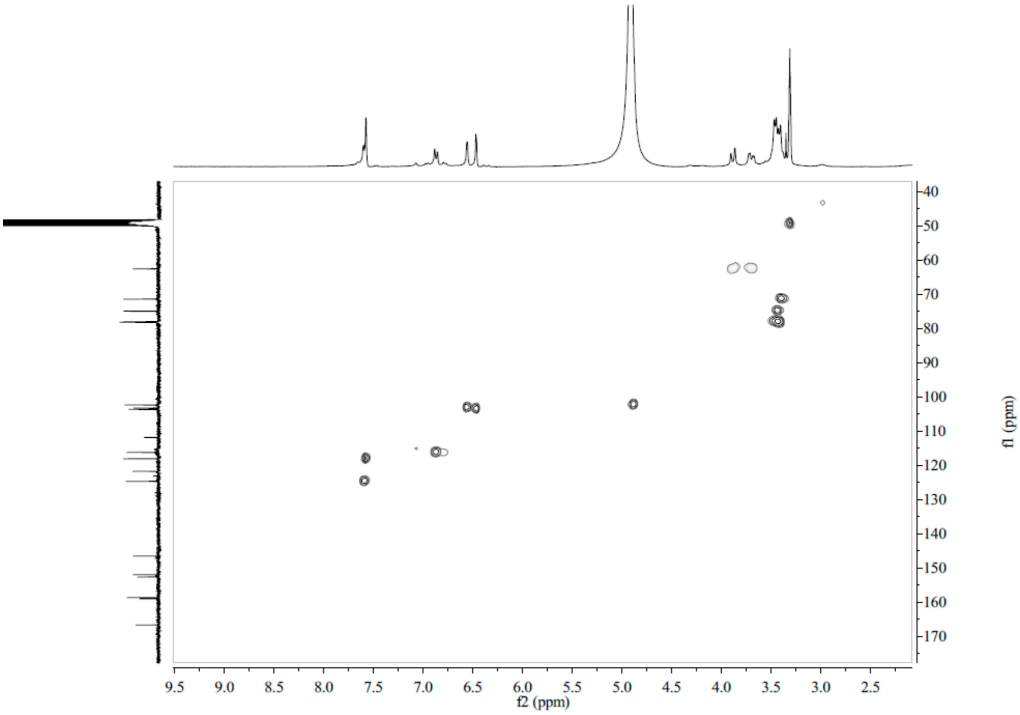

Figure S15. HSQC spectrum of **2** in CD_3OD.

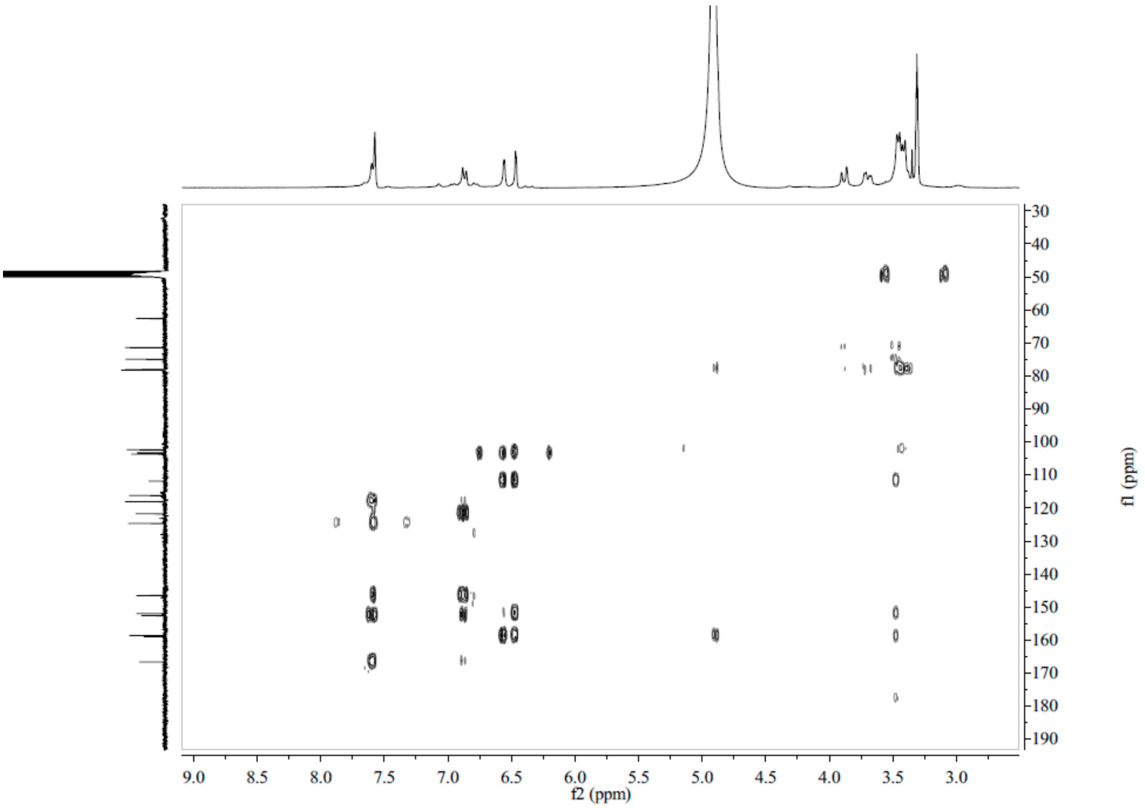

Figure S16. HMBC spectrum of **2** in CD_3OD.

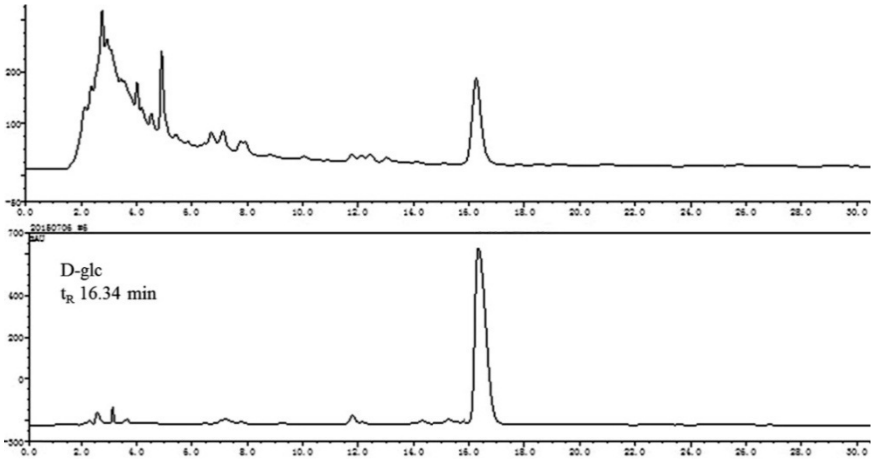

Figure S17. HPLC Analysis spectrum of sugar derivatives of **2**.

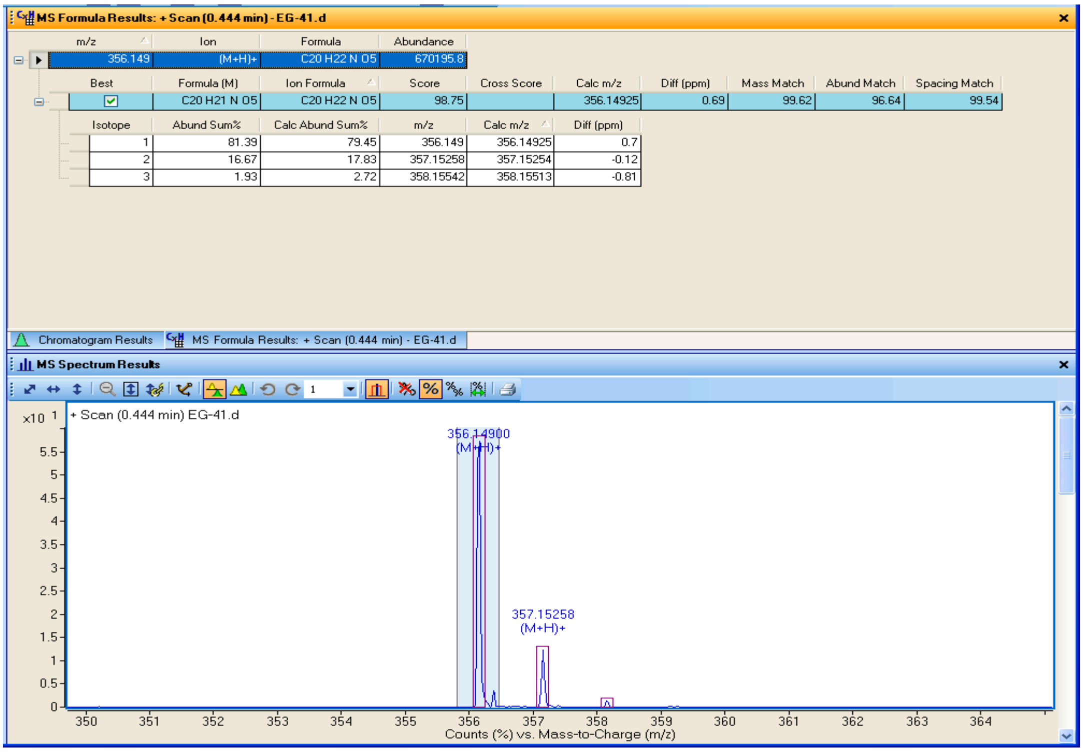

Figure S18. HR-ESI-MS spectrum of **3** (*N*-[(2*E*)-3-(4-hydroxyphenyl)-1-oxo-2-propen -1-yl]-l-tyrosine ethyl ester).

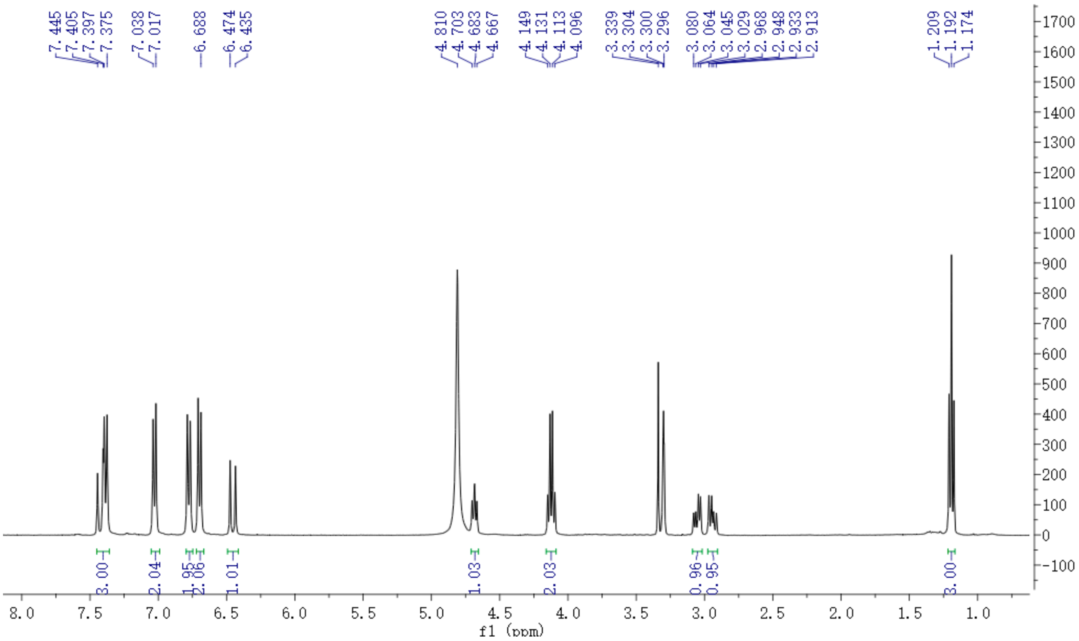

Figure S19. ^{1}H-NMR spectrum of **3** in CD_3OD.

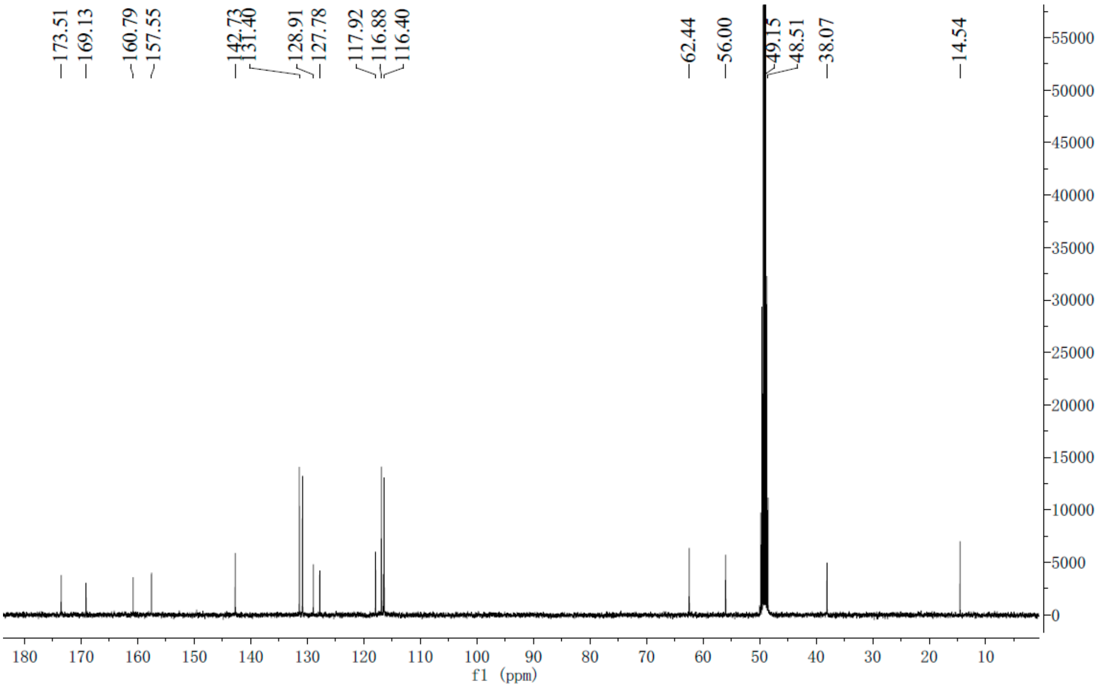

Figure S20. ^{13}C-NMR spectrum of **3** in CD_3OD.

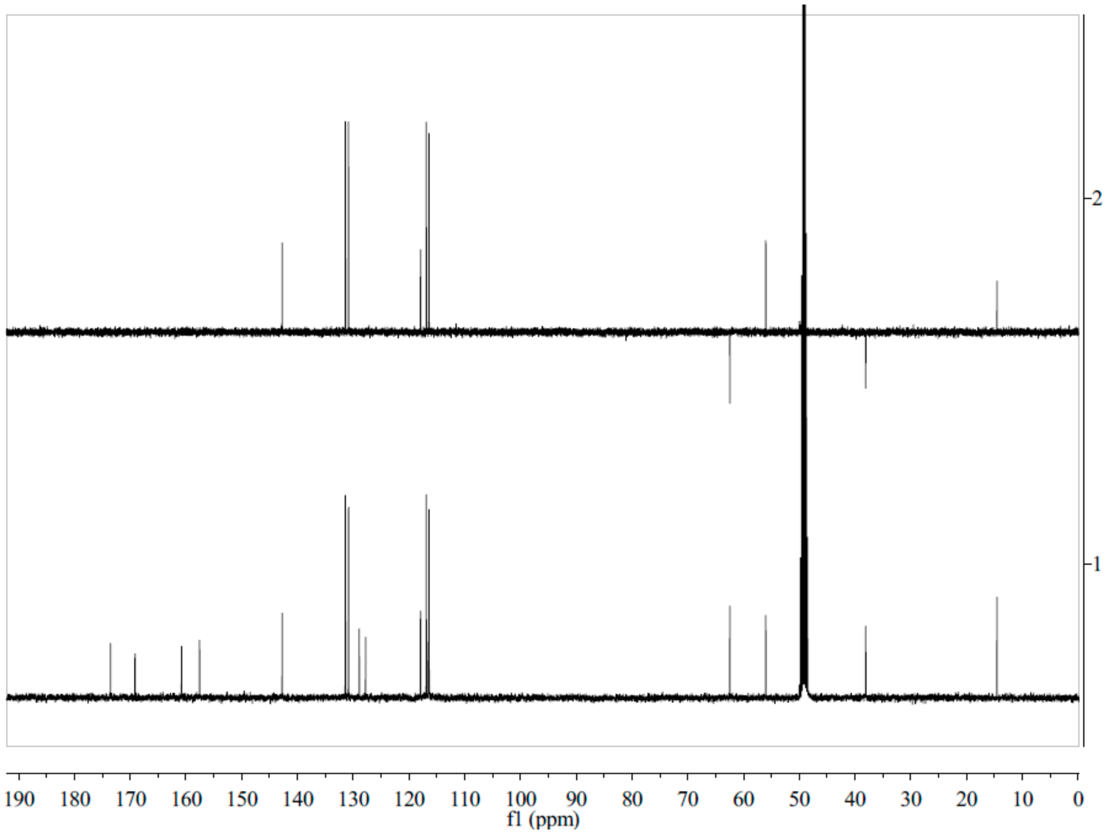

Figure S21. DEPT spectrum of **3** in CD_3OD.

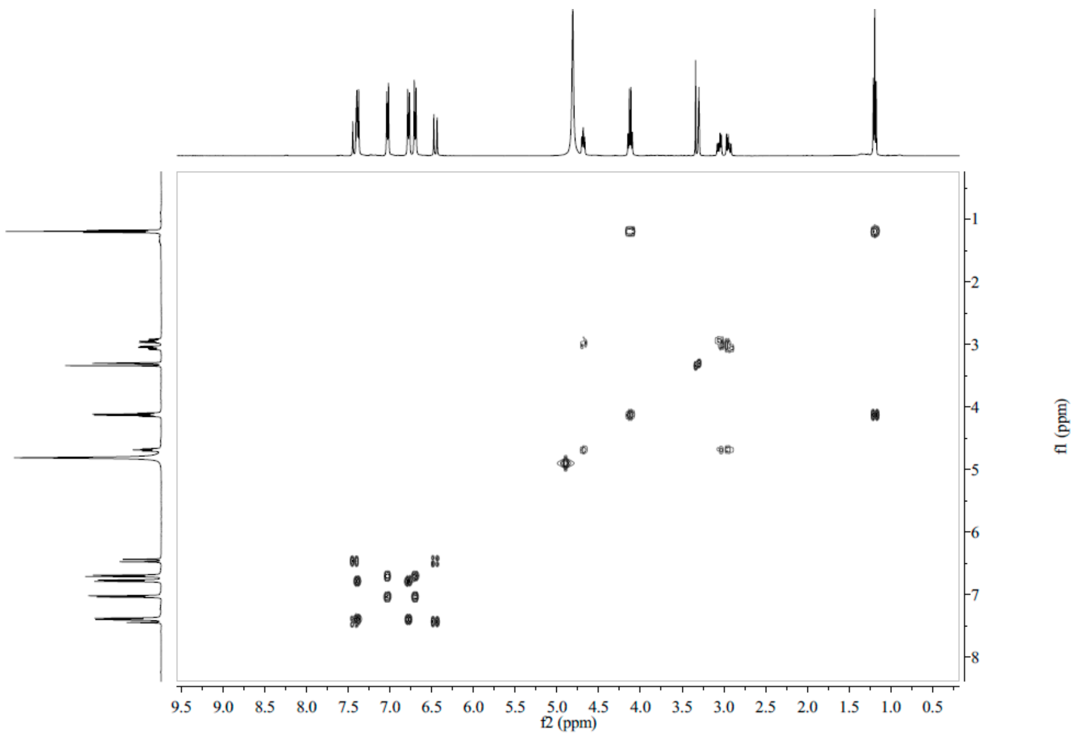

Figure S22. ^{1}H-^{1}H COSY spectrum of **3** in CD_3OD.

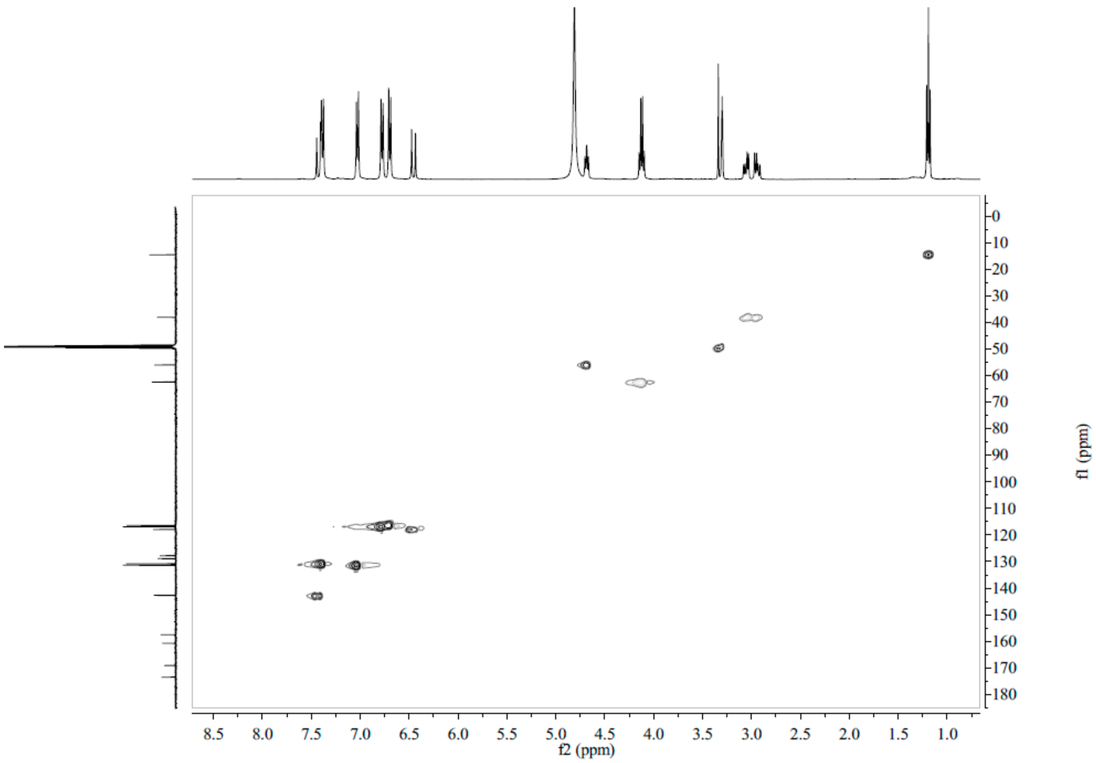

Figure S23. HSQC spectrum of **3** in CD_3OD.

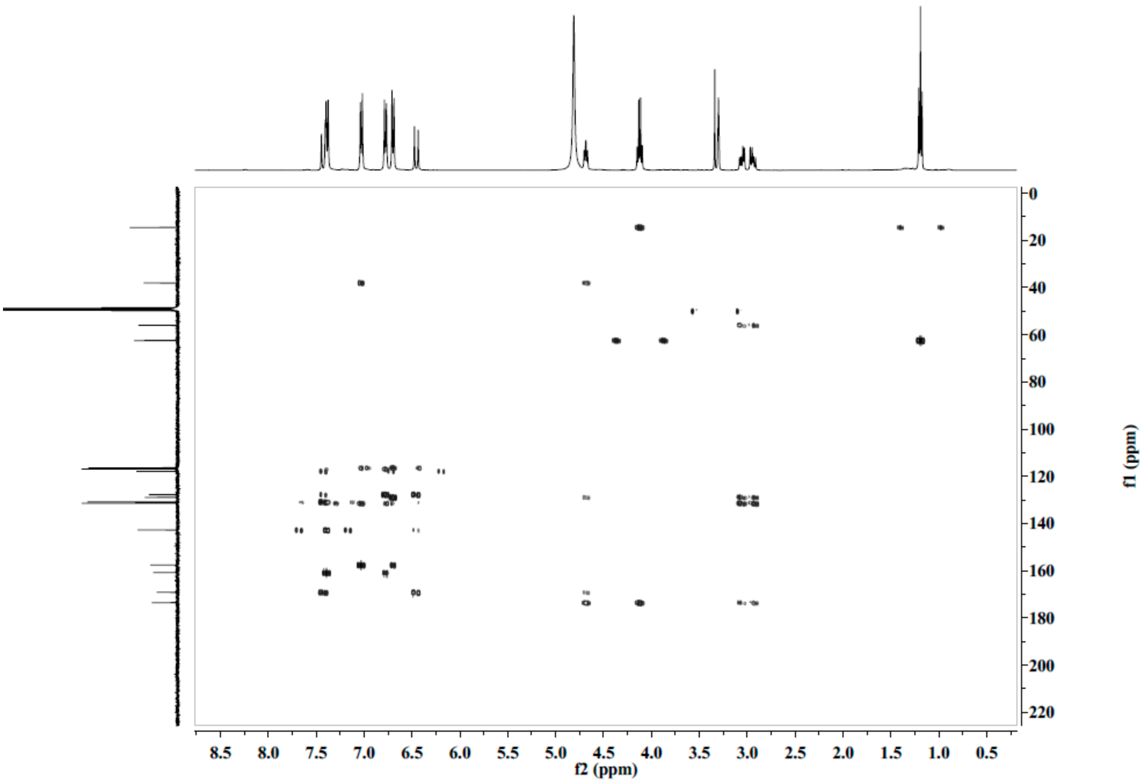

Figure S24. HMBC spectrum of **3** in CD_3OD.
